# Patterns and factors associated with pneumococcal vaccination in a prospective cohort of 1,697 patients with rheumatoid arthritis

**DOI:** 10.3389/fmed.2022.1039464

**Published:** 2023-01-09

**Authors:** Konstantinos Thomas, Argyro Lazarini, Evripidis Kaltsonoudis, Paraskevi V. Voulgari, Alexandros A. Drosos, Argyro Repa, Ainour Molla Ismail Sali, Prodromos Sidiropoulos, Panagiota Tsatsani, Sousana Gazi, Kalliopi Fragkiadaki, Maria G. Tektonidou, Petros P. Sfikakis, Pelagia Katsimbri, Dimitrios Boumpas, Evangelia Argyriou, Kyriaki A. Boki, Konstantina Karagianni, Christina Katsiari, Gerasimos Evangelatos, Alexios Iliopoulos, Eleftheria P. Grika, Panagiotis G. Vlachoyiannopoulos, Theodoros Dimitroulas, Alexandros Garyfallos, Konstantinos Melissaropoulos, Panagiotis Georgiou, Constantinos Georganas, Periklis Vounotrypidis, Konstantinos Ntelis, Maria Areti, George D. Kitas, Dimitrios Vassilopoulos

**Affiliations:** ^1^Joint Rheumatology Program, School of Medicine, National and Kapodistrian University of Athens, Athens, Greece; ^2^Rheumatology Clinic, University of Ioannina, Ioannina, Greece; ^3^Department of Clinical Immunology and Allergy, University of Crete, Heraklion, Greece; ^4^Rheumatology Unit, KAT Hospital, Athens, Greece; ^5^Rheumatology Unit, Sismanoglio Hospital, Athens, Greece; ^6^Department of Rheumatology, University of Thessaly, Larissa, Greece; ^7^Rheumatology Unit, NIMTS Hospital, Athens, Greece; ^8^4th Department of Medicine, Aristotle University of Thessaloniki, Thessaloniki, Greece; ^9^Rheumatology Unit, Agios Andreas Hospital, Patras, Greece; ^10^Private Practitioner, Athens, Greece; ^11^Private Practitioner, Thessaloniki, Greece; ^12^Private Practitioner, Kalamata, Greece; ^13^Private Practitioner, Leivadia, Greece; ^14^Hygeia Hospital, Athens, Greece

**Keywords:** rheumatoid arthritis, vaccination, biological therapy, infections, comorbidities

## Abstract

**Introduction:**

Patients with rheumatoid arthritis (RA) are at increased risk for serious infections. Pneumococcal vaccination is among the most important preventive measures, however, vaccine uptake is suboptimal. We explored the rate and factors associated with pneumococcal vaccination in a contemporary RA cohort.

**Materials and methods:**

Multi-center, prospective, RA cohort study in Greece. Patient and disease characteristics and influenza and pneumococcal vaccinations were documented at baseline and 3 years later.

**Results:**

One thousand six hundred and ninety-seven patients were included and 34.5% had already received at least one pneumococcal vaccine at baseline. Among 1,111 non-vaccinated patients, 40.1% received pneumococcal vaccination during follow-up, increasing the vaccine coverage to 60.8%. By multivariate analysis, positive predictors for pneumococcal vaccination included prescription of influenza vaccine (OR = 33.35, 95% CI: 18.58–59.85), history of cancer (OR = 2.35, 95% CI: 1.09–5.06), bDMARD use (OR = 1.85, 95% CI: 1.29–2.65), seropositivity (OR = 1.47, 95% CI: 1.05–2.05), and high disease activity (DAS28-ESR, OR = 1.33, 95% CI: 1.17–1.51). Male sex (OR = 0.65, 95% CI: 0.43–0.99) was a negative predictor for pneumococcal vaccination during follow-up.

**Discussion:**

Despite increasing rates of pneumococcal vaccine coverage, 40% of RA patients remain unvaccinated. Severe disease, bDMARD use, comorbidities, and more importantly flu vaccination were the most significant factors associated with pneumococcal vaccination, emphasizing the currently unmet need for cultivating a “vaccination culture” in RA patients.

## Introduction

Rheumatoid arthritis (RA) is the most common inflammatory arthritis in Greece and worldwide. Despite the tremendous improvement that biologic (b-) and targeted synthetic (ts-) disease-modifying antirheumatic drugs (DMARDs) have brought in its therapy, RA patients still carry a higher mortality compared to the general population (1, 2), although this gap seems to gradually close more recently.

Serious infections are considered among the most significant comorbidities of RA and contribute significantly to the excess morbidity and mortality of the disease (3). Among serious infections that require hospitalization and administration of intravenous antibiotics, pneumonia is the most common, not only in RA, but also in most rheumatic diseases. We and others have shown that respiratory tract infections are responsible for approximately half of all serious infections in RA patients (4). Their incidence is estimated to be approximately two-times higher than in the general population, similar to the one reported in patients with diabetes (5) and is especially higher in patients with RA-related interstitial lung disease (RA-ILD) (6).

Pneumonia in RA patients could be due to common bacterial pathogens such as *Streptococcus pneumoniae* or viruses such as the influenza virus (7, 8). Vaccination against these pathogens remain the most efficient measure for pneumonia prevention (9–11). Specifically for pneumococcal pneumonia, two types of pneumococcal vaccines are currently available: a 23-valent polysaccharide (PPSV23) and a 13-valent conjugate (PCV13). Despite its efficacy for the invasive forms of pneumococcal disease, PPSV23 does not provide protection against pneumonia in immunocompromised patients and those with underlying diseases (12, 13). Therefore, most guidelines suggest that patients with rheumatic diseases on immune-modifying treatments should also receive PCV13. The Greek Society of Rheumatology vaccination guidelines were published in 2016 and suggest sequential pneumococcal vaccination with both PCV13 and PPSV23 in all patients treated with conventional synthetic (cs-), ts-, or bDMARDs (14).

Despite the fact that RA was recently described as a novel risk factor for pneumonia and pneumococcal disease, vaccination uptake is low in RA patients ranging from 20 to 54% in several European and US studies indicating a significant gap in the prevention of pneumococcal disease (15, 16). There are limited data regarding the current trends in pneumococcal vaccination in RA patients.

The aim of our study was to describe the rate and factors associated with pneumococcal vaccination in a modern, large, real-life, prospective RA cohort.

## Materials and methods

### Patients

This was a multicenter, prospective study held by the RA Study Group of the Greek Rheumatology Society (ERE-EPERE) (16). Participating centers included academic and non-academic Rheumatology Clinics, National Health System outpatient clinics and private offices. Inclusion criteria included age ≥ 18 years and RA diagnosis according to ACR/EULAR 2010 classification criteria (17). During Phase 1, a cross-sectional evaluation of RA patients seen during a 9-month recruitment period was performed (June 2015–September 2016) (16). Patients were reevaluated 1 year (Phase 2) and 3 years (phase 3) later, as previously described (4, 16, 18). Participating physicians were entering data either through a printed form or electronically *via* a specific web form through a designed portal^1^ that consisted of the following sections: patient and disease characteristics, treatment patterns, and comorbidities. Data regarding influenza and pneumococcal vaccine uptake were collected at baseline retrospectively and at the 3-year follow-up time point prospectively.

Institutional Review Board (IRB) approval was provided by the Joint Rheumatology Program (Hippokration General Hospital as the co-ordinating center, 64/16-4-2015 and 7/23-3-2016) and by the local institutional boards of the following participating centers: UoI Rheumatology Clinic (6/24-3-2015), UoC Clinical Immunology and Allergy Department (1476/20-3-2012), and UoT Department of Rheumatology (42378/9-9-2015). For the remaining centers, central IRB approval was deemed sufficient. All patients provided written informed consent at the first evaluation.

### Statistical analysis

Dichotomous variables are shown as percentages and continuous variables as mean (standard deviation) for normal and median (interquartile range) for non-parametric distributions, respectively. Chi square or Fisher’s exact test was used for comparison of dichotomous and Mann–Whitney or *t*-test for continuous variables.

Multivariate logistic regression analysis was used in order to identify variables associated with pneumococcal vaccination (any type) at baseline (Phase 1) and at the end of the study (Phase 3). Variables of biologic significance and those with *p*-value < 0.1 in univariate analysis were included in the multivariate model. Variables with *p* < 0.05 were retained until the final stage of the model.

In order to adjust for the dropouts in the follow-up, we applied a sensitivity analysis with doubly robust inverse probability weighted regression (IPWR): at first, we computed inverse-probability weights from multivariable logistic regression model on dropout status (“treatment” model). We then applied the estimated inverse-probability weights to regress the outcome (administration of pneumococcal vaccination during follow-up in those patients unvaccinated at baseline). Covariates used in the “treatment” model and the “outcome” model were prespecified. This analysis produces consistent estimates because the dropout is assumed to be independent of the potential outcomes after conditioning on the “covariates.” The treatment-independent variables were chosen and included in the model according to the statistically significant differences between patients with available follow-up visit at 3 years and those lost to follow-up (seropositivity, history of arthroplasties, bDMARDs at baseline, corticosteroids at baseline, current smoking, history of serious infection, BMI, dyslipidemia).

Statistical analyses were performed with SPSS (IBM SPSS Statistics for Windows, v. 25.0. Armonk, NY, USA: IBM Corp.) and Stata 13.1 (StataCorp LCC, Texas, USA). The threshold of statistical significance was set as *p*-value < 0.05 for all comparisons.

## Results

### Patient and disease characteristics

Among 3,115 patients initially evaluated, 2,088 (67%) were available for re-evaluation 3-year later. Patient and disease characteristics were compared between these two groups and no significant differences in patient characteristics were noted (age, sex, disease duration, working status, educational level, most of comorbidities). Patients with a follow-up visit at 3 years were more likely to have features of more aggressive disease (erosions, arthroplasties, bDMARD use) with similar scores of disease activity and chronic damage (Supplementary Table 1).

**FIGURE 1:**
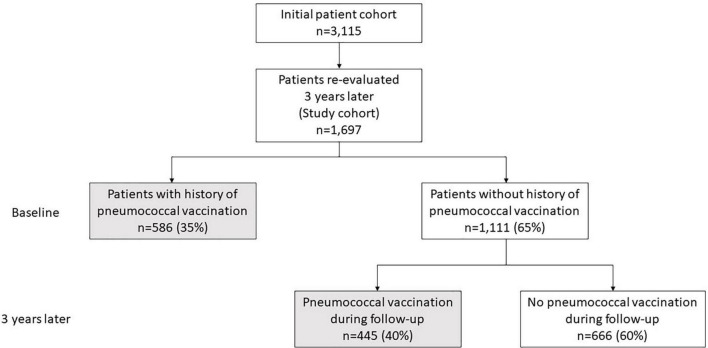
Flow chart of vaccination coverage.

Among the re-evaluated patients (*n* = 2,088), we included in the analysis those with valid information regarding the administration of any pneumococcal vaccine during the 3 years period (*n* = 1,697, 81%, Figure 1). This information was retrieved after interviewing the patients during their routine visit and reviewing their prescription data. Among those, 80.4% were women with a mean age of 61.7 years and a mean disease duration of 9.8 years (see Table 1). Approximately half were RF and/or anti-CCP positive (50.4%) with a mean DAS28-ESR of 3.41 and a median HAQ score of 0.25 at baseline. The majority were receiving csDMARDs (83.1%) while bDMARDs were used in 44.7% of patients. Approximately one third were on glucocorticoids at a mean daily prednisolone dose of 4.9 mg.

**TABLE 1:** Patient and disease characteristics at baseline.

Variable	Total
n	1,697
**Patient characteristics**	
Female, n (%)	1,358 (80.4%)
Age, years (mean, SD)	61.7 (12.6)
Disease duration, years (mean, SD)	9.8 (8.8)
Early disease (<2 years), n (%)	309 (18.2%)
RF and/or anti-CCP+, n (%)	837 (51.9%)
Erosions, n (%)	555 (44.9%)
Working, n (%)	462 (30.1%)
Tertiary education, n (%)	257 (18.4%)
**Disease characteristics**	
DAS28-ESR, mean (SD)	3.41 (1.29)
HAQ, median (SD)	0.25 (0.80)
History of arthroplasties (n, %)	171 (10.1%)
**Treatment characteristics**	
csDMARDs, n (%)	1,411 (83.1%)
bDMARDs, n (%)	759 (44.7%)
TNFi, n (%)	420 (24.7%)
Non-TNFi, n (%)	339 (20%)
Glucocorticoids, n (%)	596 (35.1%)
Glucocorticoids, mg/day, mean (SD)	4.9 (2.7)
**Comorbidities**	
Current smokers, n (%)	280 (17.7%)
Alcohol use (>1 day/week), n (%)	104 (6.5%)
Dyslipidemia, n (%)	579 (34.1%)
Coronary artery disease, n (%)	81 (4.8%)
Stroke, n (%)	50 (2.9%)
Hypertension, n (%)	724 (42.7%)
Diabetes, n (%)	234 (13.8%)
COPD and/or RA-ILD, n (%)	141 (8.3%)
Depression, n (%)	206 (12.1%)
Osteoporosis, n (%)	451 (26.6%)
Cancer (current/past), n (%)	99 (5.8%)
History of serious infection, n (%)	158 (9.3%)
History of hospitalization (last 12 months), n (%)	132 (7.8%)
BMI, kg/m^2^ (SD)	27.5 (5.2)
BMI > 30 kg/m^2^, n (%)	385 (22.7%)
RDCI, median (IQR)	0 (1)
**Vaccination history**	
Pneumococcal vaccine (ever), n (%)	586 (34.5%)
Influenza vaccine (ever), n (%)	903 (53.2%)
Influenza vaccine (last season), n (%)	535 (31.5%)

### Previous pneumococcal vaccination

At baseline, approximately one third of RA patients (*n* = 586, 34.5%) had been already vaccinated against *S. pneumoniae*. Among those patients, 344 (58.7%) received an additional dose of any pneumococcal vaccine during follow-up. We compared the patient and disease characteristics of RA patients with (*n* = 586) or without (*n* = 1,111) a history of pneumococcal vaccination at baseline (Table 2).

**TABLE 2:** Comparison of patients with and without a history of pneumococcal vaccination at baseline.

Variable	Pneumococcal vaccine (−)	Pneumococcal vaccine (+)	*P*-value
	*n* = 1,111	*n* = 586	
Female, n (%)	885 (80.1%)	473 (81%)	0.66
Age, years, mean (SD)	60.4 (13.1)	64.0 (11.2)	<0.001
Disease duration, years, mean (SD)	8.5 (8.5)	12.2 (8.9)	<0.001
Early disease (<2 years), n (%)	249 (22.4%)	60 (10.2%)	<0.001
RF and/or anti-CCP+, n (%)	518 (49.4%)	319 (56.5%)	0.007
Erosions, n (%)	306 (38.2%)	249 (57.2%)	<0.001
Working, n (%)	343 (35.1%)	119 (21.4%)	<0.001
Tertiary education, n (%)	173 (19.9%)	84 (16%)	0.06
DAS28-ESR, mean (SD)	3.46 (1.36)	3.29 (1.14)	0.015
HAQ, median (IQR)	0.25 (0.75)	0.25 (0.88)	0.39
Arthroplasties, n (%)	105 (9.5%)	66 (11.3%)	0.24
csDMARDs, n (%)	935 (84.2%)	476 (81.2%)	0.12
bDMARDs, n (%)	412 (37.1%)	347 (59.2%)	<0.001
Glucocorticoids, n (%)	374 (33.7%)	222 (37.9%)	0.08
Glucocorticoids, mg/day, mean (SD)	5.2 (2.7)	4.5 (2.7)	0.002
Current smokers, n (%)	188 (18.1%)	92 (16.8%)	0.49
Alcohol use (>1 day/week)	62 (5.9%)	42 (7.6%)	0.20
Dyslipidemia, n (%)	341 (30.7%)	238 (40.6%)	<0.001
Coronary artery disease, n (%)	46 (4.1%)	35 (6%)	0.09
Stroke, n (%)	31 (2.8%)	19 (3.2%)	0.60
Hypertension, n (%)	444 (40%)	280 (47.8%)	0.002
Diabetes, n (%)	147 (13.2%)	87 (14.8%)	0.36
COPD and/or RA-ILD, n (%)	62 (5.6%)	79 (13.5%)	<0.001
Depression, n (%)	122 (11%)	84 (14.3%)	0.04
Osteoporosis, n (%)	267 (24%)	184 (31.4%)	0.001
Cancer (current/past), n (%)	72 (6.5%)	27 (4.6%)	0.12
History of hospitalization (last 12 months), n (%)	78 (7%)	54 (9.2%)	0.11
History of serious infection, n (%)	69 (6.2%)	89 (15.2%)	<0.001
History of influenza vaccination, n (%)	347 (31.2%)	556 (94.9%)	<0.001
BMI, kg/m^2^, mean (SD)	27.2 (5.1)	28.0 (5.4)	0.005
BMI > 30 kg/m^2^, n (%)	221 (19.9%)	164 (28%)	<0.001
RDCI, median (IQR)	0 (1)	0 (1)	0.001

Those who were vaccinated were older (mean age 64 ± 11.2 vs. 60.4 ± 13.1 years, *p* < 0.001) with longer disease duration (12.2 ± 8.9 vs. 8.5 ± 8.5 years, *p* < 0.001). As for disease characteristics, vaccinated patients had more aggressive disease in terms of seropositivity (56.5 vs. 49.4%, *p* = 0.007) and erosions (57.2 vs. 38.2%, *p* < 0.001). Disease activity however was lower in the vaccinated subgroup (DAS28-ESR 3.29 ± 1.14 vs. 3.46 ± 1.36, *p* = 0.015). Regarding treatment patterns, vaccinated patients were more likely to have been treated with bDMARDs (59.2 vs. 37.1%, *p* < 0.001). Regarding co-morbidities, vaccinated patients were more likely to have dyslipidemia (40.6 vs. 30.7%, *p* < 0.001), hypertension (47.8% vs. 30%, *p* = 0.002), COPD and/or RA-interstitial lung disease (RA-ILD) (13.5 vs. 5.6%, *p* < 0.001) and BMI > 30 kg/m^2^ (28 vs. 19.9%, *p* < 0.001). Moreover, vaccinated patients were more likely to have a history of serious infections (15.2 vs. 6.2%, *p* < 0.001) and a previous vaccination for influenza (94.9 vs. 31.2%, *p* < 0.001) (Table 2).

By multivariate analysis, history of influenza vaccination (OR = 34.2, 95% CI 19.8–59.2, *p* < 0.001), previous serious infections (OR = 4.06, 95% CI 1.98–8.30, *p* < 0.001), bDMARD use (OR = 2.86, 95% CI 1.94–4.21, *p* < 0.001), longer disease duration (OR = 1.03, 95% CI 1.01–1.06, *p* = 0.005), and higher co-morbidity burden (RDCI, OR = 1.23, 95% CI 1.005–1.50, *p* = 0.04) were associated with pneumococcal vaccination (see Supplementary Table 2).

### Predictors of pneumococcal vaccination during follow-up

Since by retrospective analysis a number of unmeasured biases could have played a role, predictors of pneumococcal vaccination were evaluated prospectively in the 1,111 non-vaccinated patients. During the 2.9 years follow-up period, 445 (40.1%) more patients received a pneumococcal vaccine, increasing the cumulative coverage to 60.8% (Figure 1).

Compared to the 666 non-vaccinated patients, the vaccinated ones were older (62.6 ± 11.6 vs. 59 ± 13.9, *p* < 0.001), had more severe disease, as assessed by the higher seropositivity rate (53.3 vs. 46.9%, *p* = 0.04), higher DAS28-ESR (mean 3.75 ± 1.33 vs. 3.25 ± 1.35, *p* < 0.001), higher HAQ [median (IQR): 0.38 (0.75) vs. 0.13 (0.80), *p* = 0.03], and more frequent bDMARD use (43.4 vs. 32.9%, *p* < 0.001). Among comorbidities, prevalence of dyslipidemia, coronary artery disease, history of cancer and diabetes were numerically higher in vaccinated patients, however, only COPD and/or RA-ILD reached statistical significance (8.3 vs. 3.8%, *p* = 0.001). Similarly to the retrospective analysis, there was a significant difference in the proportion of patients who were vaccinated against the flu between vaccinated and non-vaccinated patients (96.9 vs. 42%, *p* < 0.001) (Table 3).

**TABLE 3:** Comparison of patients who were vaccinated during follow up (*n* = 445) vs. those who remained non-vaccinated (*n* = 666).

Variable	Pneumococcal vaccine (−)	Pneumococcal vaccine (+)	*P*-value
	*n* = 666	*n* = 445	
Female, n (%)	536 (81.1%)	349 (78.6%)	0.31
Age, years, mean (SD)	59 (13.9)	62.6 (11.6)	<0.001
Disease duration, years, mean (SD)	8.2 (8.2)	9 (8.8)	0.15
Early disease (<2 years)	153 (23%)	96 (21.6%)	0.58
RF and/or anti-CCP+, n (%)	300 (46.9%)	218 (53.3%)	0.04
Erosions, n (%)	174 (37.9%)	132 (38.6%)	0.84
Working, n (%)	241 (38.3%)	102 (29.2%)	0.005
Tertiary education, n (%)	123 (22%)	50 (16.2%)	0.04
DAS28-ESR, mean (SD)	3.25 (1.35)	3.75 (1.33)	<0.001
HAQ, median (IQR)	0.13 (0.80)	0.38 (0.75)	0.03
Arthroplasties, n (%)	68 (10.2%)	37 (8.3%)	0.29
csDMARDs, n (%)	553 (83%)	382 (85.8%)	0.21
bDMARDs, n (%)	219 (32.9%)	193 (43.4%)	<0.001
Glucocorticoids, n (%)	217 (32.6%)	157 (35.3%)	0.35
Glucocorticoids, mg/day, mean (SD)	5.0 (2.29)	5.5 (3.2)	0.12
Current smokers, n (%)	109 (17.7%)	79 (18.8%)	0.65
Alcohol use (>1 day/week)	37 (5.9%)	25 (6%)	0.99
Dyslipidemia, n (%)	190 (28.5%)	151 (33.9%)	0.056
Coronary artery disease, n (%)	24 (3.6%)	22 (4.9%)	0.27
Stroke, n (%)	15 (2.3%)	16 (3.6%)	0.18
Hypertension, n (%)	256 (38.4%)	188 (42.2%)	0.20
Diabetes, n (%)	82 (12.3%)	65 (14.6%)	0.27
COPD and/or RA-ILD, n (%)	25 (3.8%)	37 (8.3%)	0.001
Depression, n (%)	68 (10.2%)	54 (12.1%)	0.31
Osteoporosis, n (%)	149 (22.4%)	118 (26.5%)	0.11
Cancer (current/past), n (%)	36 (5.4%)	36 (8.1%)	0.07
History of hospitalization (last 12 months), n (%)	47 (7.1%)	31 (7%)	0.95
History of serious infection, n (%)	40 (6%)	29 (6.5%)	0.73
BMI, kg/m^2^, mean (SD)	27.1 (5.0)	27.5 (5.3)	0.21
BMI > 30 kg/m^2^, n (%)	142 (21.3%)	79 (17.8%)	0.14
RDCI, median (IQR)	0 (1)	0 (1)	0.03
Influenza vaccination during follow-up (at least 1 dose), n (%)	280 (42%)	431 (96.9%)	<0.001

**FIGURE 2:**
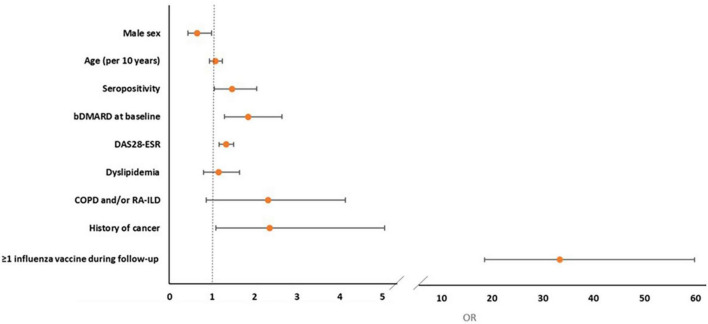
Forest plot of multivariate logistic regression analysis of factors associated with the prescription of pneumococcal vaccine during follow-up.

In multivariate logistic regression analysis, prescription of influenza vaccine (OR = 33.35, 95% CI: 18.58–59.85), history of cancer (OR = 2.35, 95% CI: 1.09–5.06), baseline bDMARD use (OR = 1.85, 95% CI: 1.29–2.65), seropositivity (OR = 1.47, 95% CI: 1.05–2.05), and high baseline disease activity (DAS28-ESR, OR = 1.33, 95% CI: 1.17–1.51) were positively associated whereas male sex (OR = 0.65, 95% CI: 0.43–0.99, *p* = 0.045) was negatively associated with pneumococcal vaccination (Table 4 and Figure 2). After the IPWR sensitivity analysis, all the above variables remained statistically significant with the exception of male sex, which was no longer significant (Supplementary Table 3).

**TABLE 4:** Uni- and multivariate logistic regression analysis of factors associated with pneumococcal vaccination among non-vaccinated patients (*n* = 1,111) during follow-up.

Variable	Univariate			Multivariate		
	OR	95% CI	*P*	OR	95% CI	*P*
Age, per 10 years	1.24	1.13–1.37	<0.001	1.08	0.94–1.24	0.27
Sex (male)	0.86	0.63–1.15	0.31	0.65	*0.43*–*0.99*	*0*.*045*
RF and/or anti-CCP positivity	1.29	1.006–1.65	0.045	*1.47*	*1.05*–*2.05*	*0*.*023*
bDMARD use at baseline	1.56	1.22–2.00	<0.001	*1.85*	*1.29*–*2.65*	*0*.*001*
DAS28-ESR at baseline	1.31	1.18–1.44	<0.001	*1.33*	*1.17*–*1.51*	<*0*.*001*
Dyslipidemia	1.29	0.99–1.66	0.056	1.15	0.80–1.65	0.44
COPD and/or RA-ILD	2.32	1.38–3.92	0.002	1.90	0.87–4.13	0.10
History of cancer	1.54	0.95–2.48	0.077	*2.35*	*1.09*–*5.06*	*0*.*03*
≥1 influenza vaccination during follow-up	6.28	5.05–7.81	<0.001	*33.35*	*18*.*58*–*59.85*	<*0*.*001*

Statistically significant values with a *p*-value <0.05 are shown in italics.

We finally sought to find in what extent the evolution of predictors for pneumococcal vaccination during follow-up could contribute in the increased vaccination coverage. We found that among the patients not on bDMARDs at neither baseline nor follow-up, 70 (4.1% of total cohort) would have reached the age cut-off of 65 years for pneumococcal vaccination in the general population. Regarding bDMARD, we estimated the bDMARD prescription in the group of patients that would not reach the 65-year age cut-off during the 3-year follow-up (that is, younger than 62 years at baseline). We found a statistically significant increase in patient on bDMARDs between the two timepoints (47.3 vs. 60.3%, *p* < 0.001), however, the respective increase in vaccination coverage was significantly higher (29 vs. 54.8%, *p* < 0.001).

### Pneumococcal vaccination coverage among patients with variable risk factors

**FIGURE 3:**
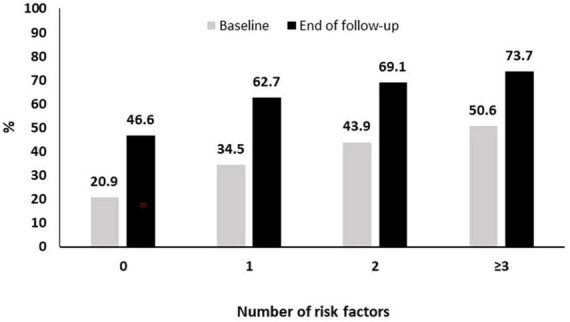
Pneumococcal vaccination coverage during follow-up according to the presence of risk factors.

As described above the pneumococcal coverage increased from 34.5% at baseline to 60.8% approximately 3 years later. This increased coverage was noted in all subgroups of patients with risk factors (current smoking, diabetes, frequent alcohol intake > 1 day/week, COPD and/or ILD, BMI < 30 Kg/m^2^, treatment with bDMARDs, coronary artery disease) for pneumococcal pneumonia. More specifically, as it can be seen in Figure 3, in patients without risk factors the coverage increased from 20.9 to 46.6% while the respective increase for those with 1, 2, or ≥3 risk factors was from 34.5 to 62.7%, 43.9 to 69.1%, and 50.6 to 73.7% (Figure 3).

## Discussion

In this prospective study, we report some novel findings regarding the contemporary pneumococcal vaccination coverage among patients with RA. First, we showed a significant increase in the cumulative rates of pneumococcal vaccination from 34.5% to more than 60% in a large prospective RA cohort (*n* = 1,697). Second, we identified the patients’ profile that was more likely to receive a pneumococcal vaccine in clinical practice. These patients had more severe disease (RF and/or anti-CCP positive, high disease activity), were treated more commonly with bDMARDs, had certain comorbidities (history of cancer) and more importantly were more likely to had been vaccinated against influenza.

In our study, the pneumococcal vaccination rate at first evaluation (34.5%) was similar to that reported in other recent RA cohorts (30–44%) (15, 19, 20). Before our study, only scarce data were available regarding the pneumococcal vaccination coverage in Greece, with one recent study that included elderly general population reporting rates of 49.5% for PCV and 23.5% for PPSV23 coverage (21). Given the suboptimal vaccine coverage, several interventions have been implemented in the literature in order to increase vaccination in patients with rheumatic diseases. Desai et al. showed that after simple and targeted interventions, the rate of patients who were up-to-date with pneumococcal vaccine coverage increased from 68 to 80% (22). The efficacy of similar measures has been also shown by others (23).

In our patient population, although they were not included in the study design, several actions were taken in parallel with the study in order to increase rheumatologists’ awareness with vaccination guidelines (24), mostly under the auspices of the Greek Rheumatology Society, that suggest the sequential vaccination with PCV13 and then with PPSV23 (both free of charge for all patients irrespective of insurance status) in unvaccinated patients receiving DMARDs. These actions included the open-access publication of Greek guidelines for vaccination of patients under DMARDs and their dissemination by a series of presentations that included rheumatology congresses, meetings with primary care physicians and workshops for rheumatology residents and post-graduate trainees in musculoskeletal health.

At the baseline evaluation, patients with longer disease duration, lower disease activity, history of serious infections and higher comorbidity burden as well as those treated with bDMARDs or vaccinated previously against the flu were more likely to have been vaccinated with at least one pneumococcal vaccine. In a similar study from the quite heterogeneous COMORA cohort, the authors found older age, absence of glucocorticoid therapy, use of bDMARD, higher educational level and country of origin to be associated with a history of PPSV23 vaccination (25). In a much smaller study from Denmark, older age and higher educational level were also found to correlate with increase pneumococcal vaccine uptake, whereas treatment type was not (26).

During the prospective period, certain factors were found to be associated with pneumococcal vaccination coverage. Seropositivity (RF and/or anti-CCP) and high disease activity (indicative of a more severe disease phenotype) were independent positive predictors of vaccination. This comes in contrast with other studies where disease activity or functional status were not associated with increased rates of pneumococcal vaccine coverage (25).

Regarding treatment patterns, in our study, RA patients on bDMARDs were most likely to get vaccinated during follow-up (OR = 1.85). Previous studies had shown discrepant results, with some showing positive association (25) while others not (26, 27).

In our study, the only comorbidity associated with increased vaccination coverage was cancer, with chronic lung disease showing a positive, albeit non-significant correlation. A previous study by Desai et al. had shown that diabetes was the only comorbidity associated with increased rates of pneumococcal vaccination (22). In the aforementioned COMORA cohort, the presence of comorbidities was associated with influenza but not pneumococcal vaccine uptake (25). Costello et al. also found that patients with more comorbidities were more likely to have been vaccinated with pneumococcal vaccine. However, they did not address the role of anti-rheumatic therapies and the type of comorbidities on pneumococcal vaccine prescription (19).

One of the most important findings of our study was that patients who got vaccinated against influenza during follow-up, had an almost 33-times higher chance of being also vaccinated against pneumococcus. A similar finding has been reported by Qendro et al. in a cohort of 312 rheumatic patients in Canada, however, with an odds ratio of 5 (27). These are important findings showing that campaigns aiming at promoting vaccination against multiple pathogens in susceptible populations such as the elderly or patients with underlying medical conditions could be successful and cost-efficient (28–30). This has been shown recently for influenza vaccination coverage during the COVID-19 pandemic (31).

We finally estimated the cumulative pneumococcal vaccination coverage among RA patients with risk factors for pneumococcal disease. We showed an increase among all subgroups with the vaccination coverage ranging from 46.6% in those without any risk factors to 73.7% in those with multiple (≥3) risk factors. Nevertheless, even among the highest risk group, one out four patients did not receive any pneumococcal vaccine during the 3 years follow-up period.

It has to be acknowledged though that the context of vaccination uptake in high-risk populations is quite complex and it is not influenced only by patient and disease characteristics, but by other factors as well (32). In a recent review, Boucher et al. identified several factors that could lead to vaccine hesitancy, such as healthcare policies, access to care, social and media influence, the understanding of vaccines’ benefits and risks, the role of healthcare professionals and the implementation of vaccination schedules (33). Unfortunately, our study could not shed more light on these important aspects.

Interestingly we found that male RA patients had a 35% lower chance to get vaccinated, despite the fact that the incidence of invasive pneumococcal disease is steadily higher in males (34, 35). It seems that vaccine uptake hesitancy could be also gender-related, as it has been recently shown with the vaccination against COVID-19 (36).

The novelty of our study lies first in the prospective evaluation and the dynamics of the vaccination coverage rates, given that most studies including RA patients are retrospective (19, 20) or cross-sectional (25). We show that even after the publication and dispersement of National Guidelines for the vaccination of patients with rheumatic diseases, the coverage falls far from optimal. Second and most important to our opinion, is our attempt to evaluate holistically the rheumatic patient as we study nearly every aspect that could influence vaccination uptake (disease severity, medications, comorbidities). Surprisingly, we report that important factors that shape the risk for severe infections such as older age and the presence of comorbidities like chronic lung disease or cardiovascular disease, were not found to influence the prescription of pneumococcal vaccine. Third, we show an association between disease activity and vaccine prescription even after adjusting for seropositivity and use of bDMARDs, in contrast to other studies with smaller number of participants (27). This is a finding that warrants further investigation.

The strengths of our study include its large size and prospective design, the long-follow-up period, the inclusion of patients seen in real-life settings and the collection of an extended dataset including most aspects of patients’ and disease characteristics. Moreover, this is one of the few studies attempting to correlate these characteristics with vaccine prescription. We believe that the interpretation of our findings could lead to significant clinical implications that will improve vaccination coverage especially in the most vulnerable patients. First, the promotion of simultaneous vaccination against multiple pathogens and the reassurance of clinicians for the effectiveness and safety of this practice will increase vaccination coverage and diminish the lost vaccine doses. Second, the addition of reminders of the need for vaccination in hard-copy or electronic medical files could accompany patients with high-risk conditions for invasive pneumococcal disease. Third, clinicians prescribing high-risk medications (DMARDS, glucocorticoids, etc.) for patients with RA or for those with co-existing high-risk comorbidities could receive automated reminders for pneumococcal and influenza vaccination.

Among its limitations we recognize that loss to follow-up is the most concerning, although it is not unusual in real-life cohorts (37). In our case, a re-evaluation at 3 years was available in two thirds of the patients while among them 81% had valid information regarding any pneumococcal vaccination. We, however, provide a sensitivity analysis with a solid methodology (IPWR) in order to adjust for the dropouts in the follow-up with similar results with our initial logistic regression analysis. Therefore, albeit important, we do not believe that loss to follow-up affected the results of our study. Moreover, we did not collect detailed information regarding the type(s) of pneumococcal vaccines administered and thus, we could not assess the degree of physicians’ and patients’ adherence to the combined vaccination schedule. Also, we did not collect data regarding the patients’ attitude toward vaccination and this could be a contributing factor in vaccine uptake. In support of this, a recent study from Greece recently showed that the belief that vaccines are not helpful, the concern for side-effects and the absence of recommendation by the treating physician were among the main reasons for non-vaccination against influenza (31). Finally, the design of the study does not allow us to estimate the specific contribution of individual variables in the reported increase in pneumococcal vaccination (vaccine hesitancy, absence of recommendation from the treating physician, increasing age, progression of disease severity, comorbidities). However, we showed that the increased vaccination coverage cannot solely attributed to age and intensification of antirheumatic therapy, but at least partially to interventions that focus on vaccine awareness.

Conclusively, our retrospective and prospective data from a contemporary, real-life, RA patient cohort show that pneumococcal vaccination coverage is gradually increasing. Despite this increase a large proportion of RA patients (∼40%) still remain unvaccinated. Among modifiable factors associated with pneumococcal vaccination, we showed that influenza vaccination was the strongest one. Thus, campaigns aiming at increasing patients’ and physicians’ awareness of vaccination benefits against multiple pathogens (influenza, pneumococcus, herpes zoster, SARS-CoV-2) could be the most efficient way to increase vaccination coverage and prevent the associated morbidity and mortality. The recent COVID-19 pandemic could also prove a decisive factor that could further increase vaccination coverage.

## Data availability statement

The datasets used and/or analyzed during the current study are available from the corresponding author on reasonable request.

## Ethics statement

Institutional Review Board (IRB) approval was provided by the Joint Rheumatology Program (Hippokration General Hospital as the co-ordinating center, 64/16-4-2015 and 7/23-3-2016) and by the local institutional boards of the following participating centers: UoI Rheumatology Clinic (6/24-3-2015), UoC Clinical Immunology and Allergy Department (1476/20-3-2012) and UoT Department of Rheumatology (42378/9-9-2015). For the remaining centers, central IRB approval was deemed sufficient. All patients provided written informed consent at first evaluation.

## Author contributions

KT and AL: conception and design of the study, statistical analysis, data interpretation, and manuscript composition. EK, AR, AS, PT, KF, PK, EA, KK, GE, EG, TD, KM, CG, PV, KN, and MA: data acquisition and data entry. PVV, PS, SG, MT, PPS, DB, KB, CK, AI, PGV, AG, and GK: data interpretation and critical revision of the manuscript. DV: conception and design of the study, data interpretation, manuscript composition, and critical revision of the manuscript. All authors read and approved the final manuscript.

## Footnotes

1
www.rheumstudygrps.gr
