# Supplementary material for: Patterns and factors associated with pneumococcal vaccination in a prospective cohort of 1,697 patients with rheumatoid arthritis
Source: Front Med (Lausanne). 2023 Jan 9;9:1039464. doi: 10.3389/fmed.2022.1039464 (PMC9868611; doi:10.3389/fmed.2022.1039464)
Supplement: Supplementary file 1 [file Data_Sheet_1.docx]

**Suppl. Table 1.Comparison of patients with or without a follow-up evaluation at the 3 year time point**

| **Variable** | **Without evaluation**  **n=1027** | **With evaluation**  **n=2088** | **p** |
| --- | --- | --- | --- |
| **Patient characteristics** |  |  |  |
| **Female, n (%)** | **812 (79.7%)** | **1662 (80%)** | **0.84** |
| **Age, years (mean, SD)** | **62.9 ±14.3** | **62.8 ±12.8** | **0.95** |
| **Disease duration, years (mean, SD)** | **7 (10.3)** | **7 (11)** | **0.29** |
| **RF and/or anti-CCP +, n (%)** | **545 (53.1%)** | **1010 (48.4%)** | **0.014** |
| **Erosions, n (%)** | **299 (37.4%)** | **658 (43.4%)** | **0.005** |
| **Working, n (%)** | **254 (28.5%)** | **536 (28.9%)** | **0.85** |
| **Tertiary education, n (%)** | **163 (18.8%)** | **293 (17.6%)** | **0.44** |
| **Disease characteristics** |  |  |  |
| **DAS28-ESR, mean (SD)** | **3.46 ±1.35** | **3.46 ±1.32** | **0.98** |
| **HAQ, median (SD)** | **0.25 (1)** | **0.25 (0.88)** | **0.85** |
| **History of arthroplasties (n, %)** | **74 (7.2%)** | **213 (10.2%)** | **0.007** |
| **Treatment characteristics** |  |  |  |
| **csDMARDs, n (%)** | **837 (81.5%)** | **1729 (82.8%)** | **0.37** |
| **bDMARDs, n (%)** | **349 (34%)** | **902 (43.2%)** | **<0.001** |
| **Glucocorticoids, n (%)** | **588 (57.3%)** | **825 (39.5%)** | **<0.001** |
| **Glucocorticoids, mg/day, mean (SD)** | **5.7 ±3.2** | **5.2 ±3.3** | **0.008** |
| **Comorbidities** |  |  |  |
| **Current smokers** | **238 (24%)** | **354 (18.1%)** | **<0.001** |
| **Alcohol use (>1 day/week)** | **53 (10%)** | **122 (12.5%)** | **0.13** |
| **Dyslipidemia** | **288 (28%)** | **711 (34.1)** | **0.001** |
| **Coronary artery disease** | **56 (5.5%)** | **118 (5.7%)** | **0.82** |
| **Stroke** | **26 (2.5%)** | **59 (2.8%)** | **0.63** |
| **Hypertension** | **386 (37.6%)** | **911 (43.6%)** | **0.001** |
| **Diabetes** | **159 (15.5%)** | **283 (13.6%)** | **0.14** |
| **COPD and/or RA-ILD** | **100 (9.7%)** | **191 (9.1%)** | **0.59** |
| **Depression** | **112 (10.9%)** | **245 (11.7%)** | **0.49** |
| **Osteoporosis** | **277 (27%)** | **581 (27.8%)** | **0.61** |
| **Cancer (current/past)** | **54 (5.3%)** | **123 (5.9%)** | **0.47** |
| **History of serious infection** | **66 (6.4%)** | **196 (9.4%)** | **0.005** |
| **History of hospitalization (last 12 months)** | **102 (9.9%)** | **170 (8.1%)** | **0.096** |
| **BMI, kg/m^2^  (SD)** | **27.05 ±4.84** | **27.5 ±5.16** | **0.017** |

**Suppl. Table 2. Uni- and multivariate logistic regression analysis of factors associated with pneumococcal vaccination at baseline (n=1.697)**

| **Variable** | **Univariate** | | | **Multivariate** | | |
| --- | --- | --- | --- | --- | --- | --- |
|  | **OR** | **95% CI** | **P** | **OR** | **95% CI** | **P** |
| **Age, per 10 years** | **1.27** | **1.17-1.37** | **<0.001** | **1.14** | **0.95-1.36** | **0.14** |
| **Sex (male)** | **1.06** | **0.82-1.36** | **0.66** | ***1.06*** | ***0.68-1.67*** | ***0.78*** |
| **Disease duration** | **1.05** | **1.03-1.06** | **<0.001** | ***1.03*** | ***1.01-1.06*** | ***0.005*** |
| **RF and/or anti-CCP positivity** | **1.33** | **1.08-1.63** | **0.007** | ***1.00*** | ***0.69-1.46*** | ***0.99*** |
| **Erosions** | **2.16** | **1.71-2.75** | **<0.001** | ***0.82*** | ***0.53-1.25*** | ***0.36*** |
| **DAS28-ESR at baseline** | **0.89** | **0.82-0.98** | **0.01** | ***0.85*** | ***0.72-1.00*** | ***0.06*** |
| **bDMARD use at baseline** | **2.46** | **2.00-3.02** | **<0.001** | **2.86** | **1.94-4.21** | **<0.001** |
| **History of serious infection** | **2.70** | **1.94-3.76** | **<0.001** | **4.06** | **1.98-8.30** | **<0.001** |
| **RDCI** | **1.41** | **1.26-1.57** | **<0.001** | ***1.23*** | ***1.005-1.50*** | ***0.04*** |
| **History of influenza vaccination** | **40.8** | **27.6-60.2** | **<0.001** | ***34.2*** | ***19.8-59.2*** | ***<0.001*** |

**Suppl. Table 3. Inverse probability weighting regression (IPWR) analysis of factors associated with pneumococcal vaccination among non-vaccinated patients during follow-up**

| **Variable** | **Multivariate** | | |
| --- | --- | --- | --- |
|  | **OR** | **95% CI** | **P** |
| **Age, per 10 years** | **1.02** | **0.85-1.20** | **0.63** |
| ***Sex (male)*** | ***1.53*** | ***0.93-2.50*** | ***0.09*** |
| **RF and/or anti-CCP positivity** | ***1.50*** | ***1.004-2.24*** | ***0.047*** |
| **bDMARD use at baseline** | ***1.82*** | ***1.17-2.84*** | ***0.008*** |
| **DAS28-ESR at baseline** | ***1.40*** | ***1.18-1.66*** | ***<0.001*** |
| **Dyslipidemia** | **1.19** | **0.76-1.88** | **0.43** |
| **COPD and/or RA-ILD** | **1.52** | **0.59-3.88** | **0.37** |
| **History of cancer** | ***2.61*** | ***1.14-5.99*** | ***0.02*** |
| **≥1 influenza vaccination during follow-up** | ***4.61*** | ***3.51-6.05*** | ***<0.001*** |

**Supplemental Tables**

**Suppl. Table 1. Comparison of baseline patient characteristics according to the follow-up status at the 3 year time point (with or without evaluation)**

RF: rheumatoid factor, anti-CCP: cyclic citrullinated peptide antibodies, DAS: disease activity score, HAQ: health assessment questionnaire, csDMARDs: conventional synthetic disease modifying anti-rrheumatic drugs, bDMARDs: biologic disease modifying anti-rheumatic drugs, TNFi: tumor necrosis factor inhibitor, nonTNFi: non-tumor necrosis factor inhibitor, COPD: chronic obstructive pulmonary disease, RA-ILD: rheumatoid arthritis-interstitial lung disease, BMI: body mass index

**Suppl. Table 2. Uni- and multivariate logistic regression analysis of factors associated with pneumococcal vaccination at baseline (n=1.697)**

OR: odds ratio, RF: rheumatoid factor, anti-CCP: cyclic citrullinated peptide antibodies, DAS: disease activity score, bDMARDs: biologic disease modifying anti-rheumatic drugs, RDCI: rheumatic disease comorbidity index

**Suppl. Table 3. Inverse probability weighting regression (IPWR) analysis of factors associated with pneumococcal vaccination among non-vaccinated patients during follow-up**

OR: odds ratio, RF: rheumatoid factor, anti-CCP: cyclic citrullinated peptide antibodies, bDMARDs: biologic disease modifying anti-rheumatic drugs, DAS: disease activity score, COPD: chronic obstructive pulmonary disease, RA-ILD: rheumatoid arthritis-interstitial lung disease
